# Preliminary Analysis of Difficulty of Importing Pattern-Based Concepts into the National Cancer Institute Thesaurus

**Published:** 2016

**Authors:** Zhe HE, James GELLER

**Affiliations:** a School of Information, Florida State University; b Department of Computer Science, New Jersey Institute of Technology

**Keywords:** Biomedical ontology, Ontology maintenance, Concept Insertion, NCIt

## Abstract

Maintenance of biomedical ontologies is difficult. We have developed a pattern-based method for dealing with the problem of identifying missing concepts in the National Cancer Institute thesaurus (NCIt). Specifically, we are mining patterns connecting NCIt concepts with concepts in other ontologies to identify candidate missing concepts. However, the final decision about a concept insertion is always up to a human ontology curator. In this paper, we are estimating the difficulty of this task for a domain expert by counting possible choices for a pattern-based insertion. We conclude that even with support of our mining algorithm, the insertion task is challenging.

## 1. Introduction

Biomedical ontologies provide a foundation in a variety of healthcare information systems [1, 2]. They have been used for encoding diagnoses, laboratory tests, problem lists in Electronic Health Records [3] and in administrative documents, e.g., for billing [4]. Moreover, with medical concepts linked by taxonomic and semantic (lateral) relationships, they also play an important role in knowledge management, data integration, and decision support [1]. The National Cancer Institute thesaurus (NCIt) contains over 100,000 concepts that are hierarchically organized in 19 distinct domains related to cancer research, e.g., neoplastic diseases, molecular abnormalities, and genes. It is a central reference terminology of NCI’s Enterprise Vocabulary Services (EVS) [5]. As new concepts are entering healthcare usage, NCIt needs to be extended as needed by its users. In Cimino’s “desiderata” [6], domain completeness is listed as the most desirable property. NCI EVS exploits internal quality assurance (QA) mechanisms as well as external participation in the QA process of NCIt.

In previous research, we have introduced a structural methodology to mine new concepts from a Unified Medical Language System (UMLS) source for inclusion in another source where they are “missing” [7, 8]. This method leverages the native term mappings of the UMLS to identify topological patterns that are indicative of a possible import. We found candidate concepts for import into SNOMED CT and domain experts confirmed the validity of this method [7, 8].

Quality assurance of the NCIt has been conducted by NCI and external researchers [5]. Min et al. constructed a partial-area taxonomy that highlighted potential errors [9]. Cohen et al. performed an automated comparative audit of the gene hierarchy of NCIt using the Entrez Gene database [10]. Mougin and Bodenreider represented the NCIt concepts in an RDF triple store to assess the consistency of the relationships among them [11]. Jiang et al. evaluated the data quality of cancer study common data elements by integrating the NCI Cancer Data Standards Repository, NCIt, and the UMLS Semantic Network with the use of tools of the Semantic Web [12].

The UMLS Metathesaurus integrates over 12 million terms from more than 170 source vocabularies into 3.1 million concepts, such that terms with the same meaning are assigned the same Concept Unique Identifier (CUI). In this paper, we apply the topological pattern-based method to recommend new concepts for the NCIt. Furthermore, we are providing an estimate of the difficulty faced by the domain experts in a concept import task, even *with* the help of our UMLS mining tool.

## 2. Methods

**Figure 1:**
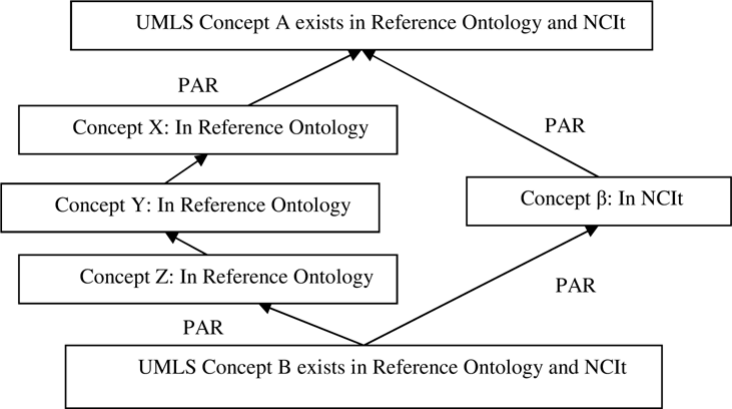
A Concept Structure in the UMLS

We are focusing on the concept structure in Figure 1. The concepts A, B, β, X, Y, and Z appear in the UMLS. A and B come from the NCIt and from a second ontology that we call the *Reference Ontology.* The concept β exists in the NCIt, but not in the Reference Ontology. The concepts X, Y and Z exist in the Reference Ontology, but not in the NCIt. The Reference Ontology is in most cases SNOMED CT, although it may also be one of several other UMLS source vocabularies. The main criteria for selecting a Reference Ontology are that it must be organized around an IS-A hierarchy backbone and must exhibit a sufficient overlap in content with the NCIt.

Looking at Figure 1, the question arises whether X or Y or Z or all of them should be considered as *missing* in the NCIt. This is not always the case. It could happen that

- X or Y or Z is a synonym of β.
- There is an error in the Reference Ontology or there is an error in the NCIt.
- β and X are alternative classifications of the concept A. For example, *Gastrointestinal Diseases* (=A) could be classified by location or disease kind. Thus, X could be *Gallbladder and biliary tree disorders*, β could be *Gastrointestinal polyps* and B could be *Polyp of gallbladder.*

The decision whether X, Y and Z are valid imports into the NCIt or whether one of the other cases applies can only be made by a medical expert with a good knowledge of the cancer domain. We note that this is a two-step decision, as explained below.

Figure 1 is approximately ◊ (diamond) shaped. (In previous work we referred to a similar structure as a “trapezoid.”) In this research, we are mining such diamond structures between the NCIt and other UMLS sources. Every such diamond *could* indicate the possible import of three concepts into the NCIt. However:

- There is a certain degree of overlap between the diamond structures, so that duplicate concepts have to be eliminated from the counting.
- As noted above, there might be alternative classifications that preclude an import.
- Even semantically valid concepts might be undesirable to the curators of NCIt, e.g., because they would be without an interesting use case for cancer researchers.
- Because of these options, the final decisions have to be made by a domain expert. In this paper, we are attempting to quantify the difficulty of the task of the expert:

How many decisions does a domain expert have to make, in the worst case, and how many choices does the expert have to select from?

### Definition 1

All concepts in a diamond between B and A that exist in the Reference Ontology, but not in the NCIt, are called *source concepts.* All concepts between B and A in the NCIt, but not in the Reference Ontology, are *target concepts.*

### Definition 2

The decision which source concepts in a diamond should be imported into the NCIt is called the *selection decision.*

### Definition 3

The decision where, in relation to existing target concepts, the selected source concepts should be located is called the *placement decision.*

### Example 1

In Figure 1, the selection decision consists of determining whether X alone, Y alone, Z alone, or a subset of X, Y and Z should be imported into the NCIt.

### Example 2

Assuming the selection decision was made that *only* Y and Z should be imported into the NCIt, the placement decision has to be made between three choices: 1) Make Y a grandparent of β and make Z a parent of β; 2) Make Y a parent of β and Z a child of β; 3) Make Y a child of β and Z a grandchild of β.

Returning to the question of how many choices a domain expert has to make in a selection decision we find the following. If the decisions are independent from each other then there are *n* decisions to be made for *n* source concepts. However, if the decisions are connected, the worst case for the number of possible choices for a selection decision (#SC) could be #*SC* = 2*^n^*. For example, in Figure 1 an expert might decide that Y is too similar to X to warrant its inclusion, but X and Z are needed.

Next we find the number of choices for the placement decision. Assume that *m* out of *n* source concepts were selected. Furthermore, we assume that there are *k* target concepts. Then the total number of placement choices (#PC) is computed by

(1) $$\#PC=\left(\underset{m}{m+k}\right)=\frac{(m+k)!}{m!*k!}$$

### Proof sketch

**Figure 2:**
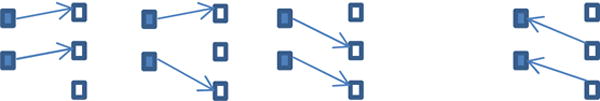
Three Possible Cases of Placement and Inversion of Last Case

After importing *m* source concepts into NCIt, when there are already *k* concepts between B and A in the NCIt, there will be a total of *m + k* concepts between B and A. Let us assume that there are *m + k* empty positions and we are assigning the *m* source concepts first to these empty positions. After this assignment, there will be *k* empty positions left unfilled. The order of the *k* target concepts is fixed, because they must be in exactly the same order as before the import, although they might be separated by imported concepts. Thus there is only one choice how to place the *k* target concepts after the *m* source concepts have been placed. Therefore, the question is reduced to how many ways there are to place the *m* source concepts in the *m+k* spaces. Refer to Figure 2 for this step for the simple case of Figure 1, with only Y and Z selected. Figure 2 contains four configurations. The three left configurations correspond to the three different possible placements.

Now we invert the direction of the arrows in Figure 2. (For space reasons, this is only done for the third configuration.). It becomes clear that the problem is equivalent to the different ways how *m* elements can be chosen from a set of *m + k* elements, which is a well-known problem in combinatorics, solved by formula (1). In this paper, we are focusing on Figure 1. However, we have discovered diamonds with eight source concepts. Hypothetically, if an expert decides that all should be imported into the NCIt, and there *would be* two target concepts then there are choices, in the worst case. For Figure 1, there are at best three and at worst eight selection choices and at most four placement choices.

(2) #PC=(108)=(10)!8!∗2!=45

## 3. Results

We discovered 769 diamonds with three source concepts and one target concept (3/1-diamonds) between SNOMED CT and the NCIt (Table 1). In total there are 812 3/1-diamonds. Each provides at least 3 selection choices for a total of 2436. Assuming 1 placement choice for each diamond, there are 2436 + 812 = 3248 possible choices. Assuming, conservatively, that it takes one minute to consider one choice, then this evaluation would take about 54 hours.

However, we have previously worked with a staff member of NCIt, and she took longer than one minute on some cases. We have informed the NCI about our research.

## 4. Discussion and Conclusions

Maintaining medical ontologies is difficult, and completely automating this task is impossible. We provide a tool that suggests which concepts should be considered for import. Staff that is knowledgeable in oncology *and* ontologies is needed for the final decisions, but such staff members are in high demand for other tasks. Furthermore, because experts sometimes disagree, at least three should be used. We have mined “diamond-shaped” concept structures from the UMLS for importing concepts into the NCIt. We have discovered 769 3/1-diamonds between SNOMED CT and the NCIt, as well as 43 3/1-diamonds with other UMLS sources. We have presented steps towards quantifying the difficulty of importing new concepts into the NCIt.

## Figures and Tables

**Table 1:** Diamond structures with three source concepts and one target concept discovered in the UMLS.

Ontology Acronym	Ontology Name	Number of 3/1 Diamonds
FMA	Foundational Model of Anatomy	15
MEDCIN	MEDCIN	19
SNOMED CT	(SNOMED was an acronym but is now a proper noun. CT = Clinical Terms)	769
Others		9
**TOTAL**		**812**
